# Lack of correlation between different congestion markers in acute decompensated heart failure

**DOI:** 10.1007/s00392-022-02036-9

**Published:** 2022-06-01

**Authors:** Svenja Haag, Alexander Jobs, Thomas Stiermaier, Carlo-Federico Fichera, Christina Paitazoglou, Ingo Eitel, Steffen Desch, Holger Thiele

**Affiliations:** 1 Department of Cardiology, Angiology and Intensive Care Medicine, University Heart Center Lübeck, Medical Clinic II, University Hospital Schleswig-Holstein, Lübeck, Germany; 2 German Center for Cardiovascular Research (DZHK), Partner Site Hamburg/Kiel/Lübeck, Lübeck, Germany; 3 Department of Internal Medicine/Cardiology, Heart Center Leipzig at University of Leipzig, Strümpellstr. 39, 04289 Leipzig, Germany; 4 Leipzig Heart Institute, Leipzig, Germany

**Keywords:** Acute decompensated heart failure, Congestion, Inferior vena cava, Dyspnea, NT-proBNP

## Abstract

**Background:**

Hospitalizations for acute decompensated heart failure (ADHF) are commonly associated with congestion-related signs and symptoms. Objective and quantitative markers of congestion have been identified, but there is limited knowledge regarding the correlation between these markers.

**Methods:**

Patients hospitalized for ADHF irrespective of left ventricular ejection fraction were included in a prospective registry. Assessment of congestion markers (e.g., NT-proBNP, maximum inferior vena cava diameter, dyspnea using visual analogue scale, and a clinical congestion score) was performed systematically on admission and at discharge. Telephone interviews were performed to assess clinical events, i.e., all-cause death or readmission for cardiovascular cause, after discharge. Missing values were handled by multiple imputation.

**Results:**

In total, 130 patients were prospectively enrolled. Median length of hospitalization was 9 days (interquartile range 6 to 16). All congestion markers declined from admission to discharge (*p* < 0.001). No correlation between the congestion markers could be identified, neither on admission nor at discharge. The composite endpoint of all-cause death or readmission for cardiovascular cause occurred in 46.2% of patients. Only NT-proBNP at discharge was predictive for this outcome (hazard ratio 1.48, 95% confidence interval 1.15 to 1.90, *p* = 0.002).

**Conclusion:**

No correlation between quantitative congestion markers was observed. Only NT-proBNP at discharge was significantly associated with the composite endpoint of all-cause death or readmission for cardiovascular cause. Findings indicate that the studied congestion markers reflect different aspects of congestion.

**Graphical abstract:**

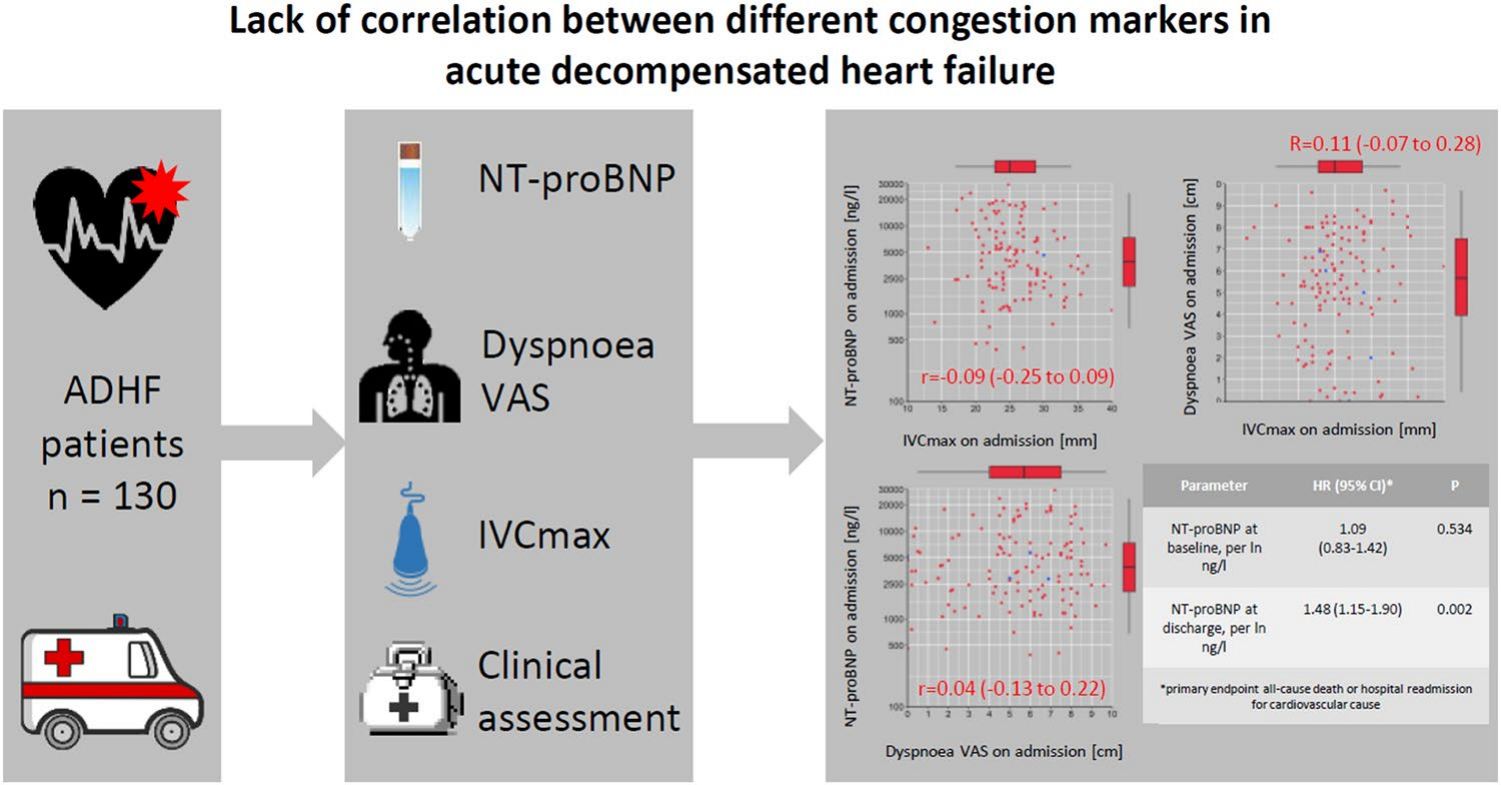

**Supplementary Information:**

The online version contains supplementary material available at 10.1007/s00392-022-02036-9.

## Introduction

Heart failure is one of the most common causes for hospital admission in Western countries including Germany [1]. Most heart failure hospitalizations take place due to acute decompensated heart failure (ADHF) with de novo or worsening signs and/or symptoms of congestion [e.g., dyspnea, rales, jugular venous distension (JVD), and edema]) [2]. Such hospitalizations have a major impact on the patient’s prognosis [3].

The clinical evaluation of patients with heart failure should include assessment for signs and symptoms associated with elevated ventricular filling pressures (“clinical congestion”). During in-hospital treatment, decongestion with the goal to achieve euvolemia is a major task for treating physicians. While signs and symptoms apparently improve during in-hospital treatment, residual signs of congestion at time of discharge predict rehospitalization and mortality remains unacceptably high after discharge. This raises the question, whether optimal decongestion was achieved at discharge or not. A prerequisite to achieve optimal decongestion is the ability to assess congestion accurately. However, prediction of elevated cardiac filling pressures by single clinical signs of congestion has a rather moderate value. Invasive assessment and monitoring of hemodynamic congestion by means of pulmonary artery catheters failed to show a benefit in the ESCAPE trial [4]. Several non-invasive markers related to the congestion status have been studied afterwards. Among other markers, change in N-terminal pro-B-type natriuretic peptide (NT-proBNP), relief of dyspnea measured on a visual analogue scale (VAS), and the inferior vena cava (IVC) diameter have been identified to be associated with the risk for rehospitalization and/or mortality [5–7].

Based on these findings, the use of a multimodal approach to assess congestion status during decongestion treatment has recently been recommended [8, 9]. However, previous studies have analyzed the prognostic impact of only a single marker. Therefore, aim of this study was to assess the correlation between different non-invasive congestion markers measured on the continuous scale and to compare their impact on prognosis in patients hospitalized for ADHF.

## Methods

### Patient population

Patients admitted to the Department of Cardiology, Angiology and Intensive Care Medicine of the University Hospital Schleswig–Holstein due to ADHF irrespective of left ventricular ejection fraction were prospectively enrolled in a single center registry. ADHF was defined as heart failure-related functional limitation (New York Heart Association [NYHA] class ≥ II) and peripheral edema or pulmonary congestion (defined as either rales on lung auscultation or pulmonary congestion on chest X-ray). No specific exclusion criteria were applied. All patients provided written informed consent. The study was approved by the local ethics committee of the University of Lübeck and all patients gave written informed consent.

### Congestion markers

Congestion was assessed at predefined visits using different non-invasive markers. More specific, we recorded clinical signs by physical examination, NT-proBNP, IVC diameters measured during focused echocardiography studies using a handheld device (Vscan Extend™, GE Healthcare, Chicago, Illinois, United States) at bedside, and dyspnea using a VAS at hospital admission and hospital discharge.

Bedside IVC measurements were performed as recommended in current echocardiography guidelines. In short, the IVC was visualized in the subcostal view and measured just before the entrance of hepatic veins. IVC_max_ is defined as the maximum diameter at end-exspiration [10]. Dyspnea was assessed using a 10 cm VAS. Patients marked their degree of dyspnea on the DVAS (0 cm = no dyspnea; 10 cm = worst imaginable dyspnea). A clinical congestion score incorporated several clinical congestion signs (Table 1) similar to the EVEREST congestion score whose elevated values are significantly associated to an increased risk of all-cause-mortality and heart failure hospitalization [11].

**Table 1:** Clinical congestion score

Signs	Points			
	0	1	2	3
JVP	< 6 cmH_2_O	6–10 cmH_2_O	> 10 cmH_2_O	
Rales	none	< 1/3	1/3–2/3	> 2/3
Edema	0	1 +	2 +	3 +

The clinical congestion score is the sum of the degree of congestion in each domain. The degree of congestion in each domain is rated as follows: edema: 0 = none edema, 1 +  = few (ankle or shank edema), 2 +  = moderate (ankle and shank edema), 3 +  = severe (peripheral edema up to anasarca); rales: 0 = no rales present in pulmonary auscultation, < 1/3 = moist rales present in the lower 1/3 of one or both lungs in pulmonary auscultation, 1/3–2/3 = moist rales present in the lower 1/3 up to lower 2/3 of one or both lungs in pulmonary auscultation, > 2/3 = present of moist rales all over both lungs during pulmonary auscultation; jugular venous pressure (JVP): < 6 cmH_2_O = absence of jugular venous distension (JVD) above the clavicle and no hepatojugular reflux (HJR), 6–10 cmH_2_O = JVD less than 4 cm above the clavicle and at most mild enlargement with HJR, > 10 cmH_2_O = JVD $\ge$ 4 cm above the clavicle and considerable enlargement with HJR

### Follow-up and outcome

Follow-up visits were performed at 180 and 360 days after discharge by means of structured telephone interviews. The primary endpoint was the composite of all-cause death or readmission for cardiovascular cause. Length of hospital stay was analyzed as secondary endpoint.

### Statistical analyses

We calculated summary statistics for patient characteristics and outcomes stratified by the occurrence of a primary endpoint event as median with interquartile range (IQR) for continuous variables and frequencies with percentages for categorical variables. Group differences were tested using Kruskal–Wallis or Chi-square tests, respectively. All congestion markers are continuous and at least ordinal. We assumed monotonic relations between each pair of congestions markers. Therefore, Spearman’s correlation coefficients were calculated to assess the degree of correlation between pairs of congestion markers. Cox regression analyses were performed to study the association of continuous congestion markers with the primary endpoint. In addition to univariable models, multivariable models considering all congestion markers were created to identify independent predictors. Linear regression analyses were performed to study the association of continuous congestion markers with the length of hospital stay. Length of hospital stay and NT-proBNP were relatively skewed and therefore log-transformed for statistical analyses. Missing data were handled with multiple imputation using chained equations (number of imputations = 5). All analyses were performed using R (R Foundation for Statistical Computing, Vienna, Austria).

## Results

In total, 130 patients were prospectively enrolled from November/2016 to September/2019. Baseline characteristics of the study population by outcome status are shown in Table 2. Patients with an outcome event during follow-up were older than patients without an outcome event (83 [IQR 76; 86] years versus 78 [IQR 70; 85] years, *p* = 0.021). However, other baseline characteristics including medical history and echocardiography findings did not differ between both the groups.

**Table 2:** Patient characteristics

	All patients	Patients without event	Patients with event	*p*-value
	*n* = *130*	*n* = *70*	*n* = *60*	
Age, years	80 [73;85]	78 [70;85]	83 [76;86]	0.021
Female sex	41 (31.5%)	23 (32.9%)	18 (30.0%)	0.873
BMI, kg/m^2^	28.5 [25.7;32.1]	28.9 [26.4;32.9]	28.0 [25.5;31.2]	0.231
SBP, mmHg	132 [120;150]	130 [120;153]	132 [120;150]	0.578
Pulse, min^−1^	83 [70;99]	83 [72;101]	82 [65;98]	0.482
eGFR, ml/min/1.73 m^2^	52 [39;65]	52 [43;70]	50 [37;61]	0.091
Comorbidities and medical history				
Atrial fibrillation	64 (49.2%)	35 (50.0%)	29 (48.3%)	0.989
Ischemic heart disease	70 (53.8%)	35 (50.0%)	35 (58.3%)	0.439
PCI	55 (42.3%)	27 (38.6%)	28 (46.7%)	0.451
CABG	29 (22.3%)	13 (18.6%)	16 (26.7%)	0.371
Anemia	70 (53.8%)	34 (48.6%)	36 (60.0%)	0.260
Hyponatremia	16 (12.3%)	9 (12.9%)	7 (11.7%)	> 0.99
Echocardiography				
HFrEF	61 (46.9%)	30 (42.9%)	31 (51.7%)	0.408
EF, %	40 [30;53]	40 [30;53]	38 [30;51]	0.250
Mitral regurgitation				0.406
None	27 (20.8%)	17 (24.3%)	10 (16.7%)	
I	47 (36.2%)	21 (30.0%)	26 (43.3%)	
II	39 (30.0%)	23 (32.9%)	16 (26.7%)	
III	17 (13.1%)	9 (12.9%)	8 (13.3%)	
Tricuspid regurgitation				0.753
None	28 (21.5%)	17 (24.3%)	11 (18.3%)	
I	38 (29.2%)	21 (30.0%)	17 (28.3%)	
II	36 (27.7%)	19 (27.1%)	17 (28.3%)	
III	28 (21.5%)	13 (18.6%)	15 (25.0%)	

Patient characteristics based on the first imputed dataset. Values are median (interquartile range) or number of patients (%). Anemia was defined according to the World Health Organization as hemoglobin levels < 12.0 g/dl in women and < 13.0 g/dl in men. Hyponatremia was defined as sodium levels < 135 mmol/l. *BMI* body mass index, *CABG *coronary artery bypass graft, *eGFR* estimated glomerular filtration rate, *LVEF* left ventricular ejection fraction, *PCI *percutaneous coronary intervention, *SBP* systolic blood pressure

### Trajectory of and correlation between congestion markers

**Fig. 1:**
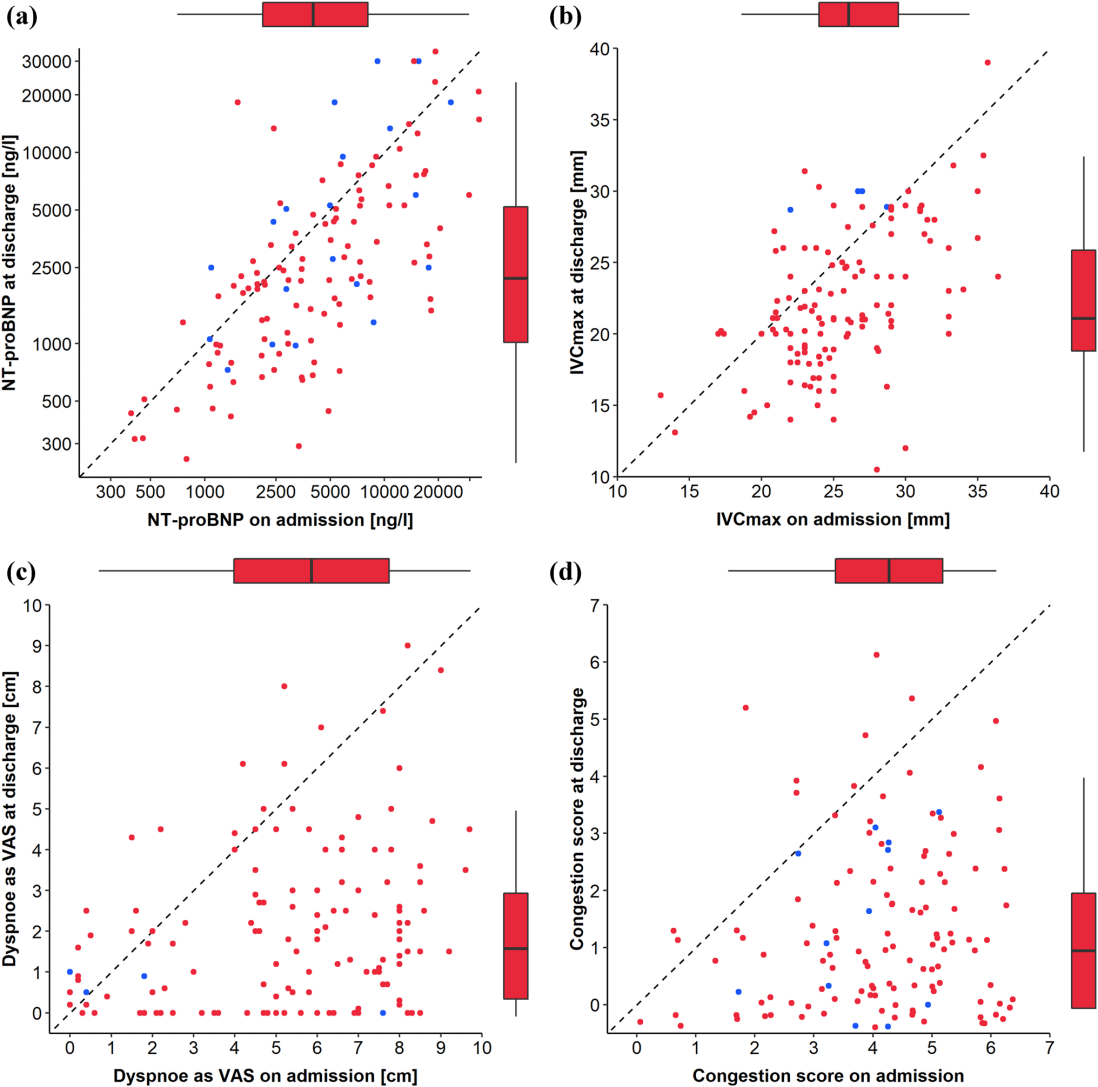
Scatter plots with overlaying jitter plots visualizing the distribution of congestion markers on admission and at discharge. Red points indicate measurements based on observed data and blue points indicate measurements based on imputed data. N-terminal pro–B-type natriuretic peptide (NT-proBNP), maximum inferior vena cava diameter (IVCmax), dyspnea as visual analogue scale (VAS), and the clinical congestion score declined from admission to discharge as visualized by the location of the majority of point in the lower right part of the plots (*P* < 0.001 for all by Wilcoxon signed rank test)

**Fig. 2:**
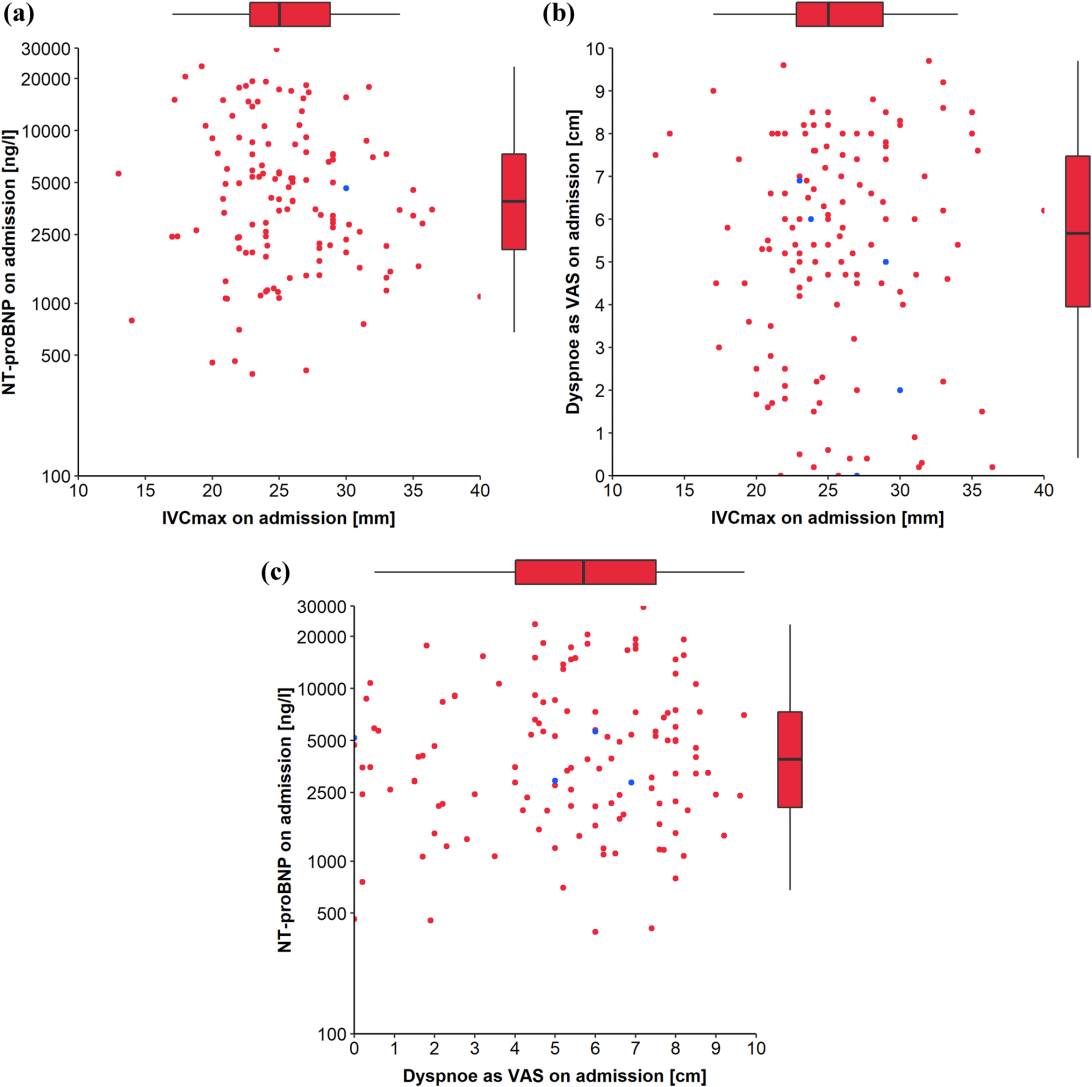
Scatter plots visualizing correlations between congestion markers on admission **a** N-terminal pro–B-type natriuretic peptide (NT-proBNP) versus maximum inferior vena cava diameter (IVCmax); **b** dyspnea as visual analogue scale (VAS) versus IVCmax; **c** NT-proBNP versus dyspnea as VAS. Red points indicate measurements based on observed data and blue points indicate measurements of which at least one value was imputed. Box plots summarize distribution of the corresponding congestion marker

Measures of all objective congestion markers declined over the course of in-hospital treatment (Fig. 1). Neither on hospital admission nor at hospital discharge did objective congestion markers correlate with each other as shown by visual inspection of scatterplots and correlation analyses (Table 3 and Fig. 2). NT-proBNP was not different at admission (3966 [IQR 2311; 7087] ng/l versus 4040 [2087; 8638] ng) nor at discharge (2116 [1024; 4340] ng/l versus 2136 [813; 4925] ng) between patients with and without atrial fibrillation.

**Table 3:** Congestion on admission and at discharge

	All patients	Patients without event	Patients with event	*p*-value
	*n* = *130*	*n* = *70*	*n* = *60*	
Admission				
NYHA class				0.839
II	4 (3.08%)	2 (2.86%)	2 (3.33%)	
III	84 (64.6%)	47 (67.1%)	37 (61.7%)	
IV	42 (32.3%)	21 (30.0%)	21 (35.0%)	
Rales				0.281
Absent	23 (17.7%)	15 (21.4%)	8 (13.3%)	
< 1/3	70 (53.8%)	33 (47.1%)	37 (61.7%)	
1/3–2/3	36 (27.7%)	21 (30.0%)	15 (25.0%)	
> 2/3	1 (0.77%)	1 (1.43%)	0 (0.00%)	
Edema				0.205
Absent	4 (3.08%)	4 (5.71%)	0 (0.00%)	
1 +	32 (24.6%)	17 (24.3%)	15 (25.0%)	
2 +	54 (41.5%)	31 (44.3%)	23 (38.3%)	
3 +	40 (30.8%)	18 (25.7%)	22 (36.7%)	
Jugular venous pressure				0.815
< 6 cm H_2_O	35 (26.9%)	19 (27.1%)	16 (26.7%)	
6–10 cm H_2_O	20 (15.4%)	12 (17.1%)	8 (13.3%)	
> 10 cm H_2_O	75 (57.7%)	39 (55.7%)	36 (60.0%)	
Congestion score	5.00 [4.00;5.75]	4.00 [3.00;6.00]	5.00 [4.00;5.00]	0.481
IVCmax, mm	25 [23;29]	25 [23;27]	25 [23;29]	0.276
DVAS, cm	5.6 [3.7;7.5]	5.5 [4.3;7.5]	5.8 [2.5;7.5]	0.848
NT-proBNP, ng/l	4011 [2103;8107]	3904 [2103;6981]	4585 [2132;9528]	0.422
Discharge				
NYHA class				0.571
I	30 (23.1%)	17 (24.3%)	13 (21.7%)	
II	61 (46.9%)	35 (50.0%)	26 (43.3%)	
III	35 (26.9%)	17 (24.3%)	18 (30.0%)	
IV	4 (3.08%)	1 (1.43%)	3 (5.00%)	
Rales				0.463
Absent	100 (76.9%)	56 (80.0%)	44 (73.3%)	
< 1/3	21 (16.2%)	11 (15.7%)	10 (16.7%)	
1/3–2/3	9 (6.92%)	3 (4.29%)	6 (10.0%)	
Edema				0.264
Absent	77 (59.2%)	46 (65.7%)	31 (51.7%)	
1 +	43 (33.1%)	20 (28.6%)	23 (38.3%)	
2 +	10 (7.69%)	4 (5.71%)	6 (10.0%)	
Jugular venous pressure				0.320
< 6 cm H_2_O	94 (72.3%)	53 (75.7%)	41 (68.3%)	
6–10 cm H_2_O	12 (9.23%)	4 (5.71%)	8 (13.3%)	
> 10 cm H_2_O	24 (18.5%)	13 (18.6%)	11 (18.3%)	
Congestion score	1.00 [0.00;2.00]	1.00 [0.00;2.00]	1.00 [0.75;2.00]	0.011
IVCmax, mm	21 [19;26]	21 [19;25]	21 [19;27]	0.404
DVAS, cm	1.8 [0.4;3.0]	1.8 [0.5;2.7]	1.7 [0.1;3.3]	0.903
NT-proBNP, ng/l	2160 [988;5283]	1826 [794;3291]	2992 [1707;7352]	0.002

Congestion markers on admission and at baseline based on the first imputed dataset. Values are median (interquartile range) or number of patients (%). *IVCmax* maximum inferior vena cava diameter, *NT-proBNP* N-terminal pro–B-type natriuretic peptide, *NYHA*  New York Heart Association, *DVAS * visual analogue scale for dyspnea

### Congestion markers and length of hospital stay

Length of hospital stay was in median 9 days (IQR 6 to 16 days). Length of hospital stay did not differ between patients with and without a primary outcome event (*P* = 0.58 by Wilcoxon rank sum test). Of the congestion markers, only the clinical congestion score and IVCmax at baseline were significantly associated with length of hospital stay (Online Table 1).

### Congestion markers and the combined risk for death or readmission

During the median follow-up of 188 (IQR 57–212) days, 60 of 130 (46.2%) patients suffered the composite primary outcome event of all-cause death or hospital readmission for cardiovascular cause. In total, 34 of 130 (26.2%) patients died during follow-up.

In clinical routine, severity of heart failure is generally assessed using the NYHA classification and severity of congestion by clinical signs and symptoms. Neither at admission nor at discharge did the NYHA class differ between patients with and without an outcome event in our study population (Table 4). Likewise, no recorded measure of congestion differed between patients with and without an outcome event at admission. However, patients with an outcome event had more severe congestion as measured by the clinical congestion score and NT-proBNP at discharge (Table 4).

**Table 4:** Correlation between congestion markers on admission and at discharge

Hospital admission				
	CS	IVCmax	VAS	NT-proBNP
CS		0.09 (− 0.08 to 0.26)	0.04 (− 0.14 to 0.22)	− 0.07 (− 0.24 to 0.101)
IVCmax	0.09 (− 0.08 to 0.26)		0.11 (− 0.07 to 0.28)	− 0.09 (− 0.25 to 0.09)
VAS	0.04 (− 0.14 to 0.22)	0.11 (− 0.07 to 0.28)		0.04 (− 0.13 to 0.22)
NT-proBNP	− 0.07 (− 0.24 to 0.101)	− 0.09 (− 0.25 to 0.09)	0.04 (− 0.13 to 0.22)	

Hospital discharge				
	CS	IVCmax	DVAS	NT-proBNP
CS		0.17 (− 0.01 to 0.34)	0.02 (− 0.16 to 0.2)	0.17 (− 0.02 to 0.347)
IVCmax	0.17 (− 0.01 to 0.34)		0.12 (− 0.06 to 0.29)	0.05 (− 0.13 to 0.24)
DVAS	0.02 (− 0.16 to 0.2)	0.12 (− 0.06 to 0.29)		0.05 (− 0.13 to 0.23)
NT-proBNP	0.17 (− 0.02 to 0.347)	0.05 (− 0.13 to 0.24)	0.05 (− 0.13 to 0.23)	

Spearman correlation coefficients and respective 95% confidence intervals for congestion marker measures at hospital admission and at hospital discharge based on multiple imputed data. *CS* clinical congestion score, *IVCmax* maximum inferior vena cava diameter, *NT-proBNP*  N-terminal pro-B-type natriuretic peptide, *DVAS*   visual analogue scale for dyspnea

None of the congestion markers measured at admission was associated with the primary outcome (Table 5). Higher NT-proBNP levels at discharge were, however, predictive for a primary outcome event during follow-up. Dichotomizing discharge NT-proBNP values revealed that an NT-proBNP level < 1.500 ng/l was also associated with better outcome in our study population (Table 5).

**Table 5:** Association of congestion markers with clinical outcome

	HR (95% CI)	*P*	FMI
Admission			
*Univariable models*			
NT-proBNP, per ln ng/l	1.09 (0.83–1.42)	0.534	0
IVCmax, per mm	1.03 (0.97–1.09)	0.373	0.005
CS, per point	1.11 (0.94–1.32)	0.223	0.005
DVAS, per cm	0.95 (0.86–1.05)	0.332	0.025
DVAS < median	0.96 (0.57–1.61)	0.876	0.021
Discharge			
*Univariable models*			
NT-proBNP, per ln	1.48 (1.15–1.90)	0.002	0.170
NT-proBNP < 1,500 ng/l	0.45 (0.22–0.91)	0.026	0.168
IVCmax, per mm	1.01 (0.96–1.06)	0.627	0.029
CS, per point	1.17 (0.97–1.4)	0.093	0.144
CS < 2 points	0.63 (0.36–1.11)	0.112	0.165
DVAS, per cm	0.97 (0.85–1.11)	0.645	0.009
DVAS < median	1.14 (0.67–1.92)	0.628	0.023
*Multivariable models*			
NT-proBNP, per ln	1.47 (1.13–1.90)	0.004	0.188
+ IVCmax, per mm	1.00 (0.95–1.06)	0.915	0.097
+ CS, per point	1.13 (0.93–1.37)	0.235	0.199
+ DVAS, per cm	0.96 (0.83–1.10)	0.529	0.012
NT-proBNP < 1,500 ng/l	0.45 (0.22–0.91)	0.027	0.166
+ IVCmax < 21 mm	1.03 (0.61–1.76)	0.899	0.058
+ CS < 2 points	0.64 (0.36–1.14)	0.128	0.190
+ DVAS < median	1.09 (0.64–1.84)	0.757	0.013

Risk for all-cause death or readmission for cardiovascular cause depending on the degree of congestion as measured by quantitative congestion markers. *CI *confidence interval, *CS* clinical congestion score, *FMI *fraction of missing information, *HR* hazard ratio, *IVCmax *maximum inferior vena cava diameter, *NT-proBNP*  N-terminal pro-B-type natriuretic peptide, *DVAS *visual analogue scale for dyspnea

Considering all congestion markers in a multivariable model identified only NT-proBNP at discharge as independent predictor for the primary clinical outcome. This finding was true for continuously coded as well as dichotomized congestion marker variables (Table 5).

In line with this, the multivariable model considering all congestion markers measured at discharge did not predict the primary outcome better than a univariable model considering only the NT-proBNP level at discharge. Again, this finding was persistent for continuously coded as well as dichotomized congestion marker variables (Table 5).

## Discussion

The main findings of our study are as follows: (1) the investigated non-invasive congestion markers do not correlate with each other and (2) NT-proBNP levels may predict outcome after ADHF hospitalization.

ADHF is a sentinel event in patients with chronic heart failure because the mortality risk considerably increases after such hospitalization. Hospital presentation is generally triggered after rise of clinical signs and symptoms of congestion above a bearable level. This clinical manifestation (i.e., clinical congestion) of elevated filling pressures is, however, the only visible part during clinical examination. Detection of clinical congestion can be challenging, crude, and operator-dependent. While no sign has perfect predictive value, signs of congestion in aggregate are useful to understanding the hemodynamic status and can inform treatment decisions. In patients without or after resolution of typical signs of congestion, elevated cardiac filling pressures (i.e., hemodynamic congestion) and elevated pressure in the venous system may persists. Previous studies found that filling pressures start to rise days to weeks before hospital presentation for ADHF [12]. Sensitivity and specificity of clinical signs and symptoms to detect hemodynamic congestion are low [13, 14]. Therefore, invasive measurement of right atrial pressure and pulmonary capillary wedge pressure (PCWP) by means of pulmonary artery catheterization remain the reference standard for evaluation of hemodynamic congestion [15]. However, this invasive monitoring is technically demanding, prone to complications, and did not improve outcome in the ESCAPE trial with pulmonary artery catheters being in place only for a median of 1.9 days [4]. As a result, the optimal approach to assess congestion and guide treatment during hospitalization for ADHF remains unknown and challenging. This is why non-invasive congestion markers such as NT-proBNP, IVC diameters, B-lines in lung ultrasound, thoracic impedance, and intra-abdominal pressure have been examined in terms of their prognostic relevance in recent years. For example, patients with chronic heart failure free of clinical signs of congestion and only mild symptoms (i.e., NYHA ≤ II) often had evidence of congestion on ultrasound in an observational study. These patients were at risk for clinical events, highlighting the importance of sub-clinical congestion [16, 17].

We assessed NT-proBNP, IVCmax, DVAS, and clinical congestion signs in patients enrolled in a prospective ADHF registry. All these congestion markers individually have been proven to be of prognostic importance in previous studies [5, 6, 18–22]. However, correlations between these markers and their relative prognostic importance compared to each other are largely unknown. Despite missing prospective studies, recently published position papers recommend a multi-parameter-based approach to evaluate decongestion and to avoid early readmission [8, 9].

Each congestion marker has specific advantages and limitations. NT-proBNP is the most thoroughly studied biomarker in heart failure. It is a quantitative marker and a strong independent predictor for death and readmission of cardiovascular cause in ADHF patients [7]. For example, a NT-proBNP level < 1,500 ng/l at discharge has previously been identified to signify a low risk of death in patients with ADHF [7]. Furthermore, NT-proBNP has a very high negative predictive value for ruling out ADHF as diagnosis in patients presenting with dyspnea [23]. However, guiding in-hospital treatment of ADHF patients based on NT-proBNP values failed to improve 6-month outcome in the PRIMA II trial [24]. In addition, increased NT-proBNP levels are non-specific for heart failure. Impaired renal function and pulmonary embolism are for example associated with elevated NT-proBNP-levels. Furthermore, cardiac distress, which occurs in sepsis or pulmonary disease, can lead to elevation of natriuretic peptides. Nevertheless, we found higher NT-proBNP values at discharge to be associated with a primary outcome event (death or cardiovascular hospitalization) in our study, supporting previous findings in a pooled analysis of ADHF registries [24]. However, NT-proBNP measured at admission was not predictive as previously shown for B-type natriuretic peptide in a post-hoc analysis of the VERITAS trial [25].

The IVC diameter can be used to estimate right atrial pressure semiquantitatively [10, 26]. After a short period of training, the IVC diameter can easily and reliably be determined even with a handheld ultrasound device at bedside [27–32]. However, visualization of the IVC may not be possible in around 15% of cases due to anatomical factors such as overweight or meteorism [33]. Other factors such as positive pressure ventilation, sex, and height may also influence the IVC diameter independently of the right atrial pressure [34–36]. Previous studies identified IVCmax measured at hospital admission as predictor for all-cause mortality in ADHF populations [18, 21]. However, we observed no association of IVCmax with the primary outcome of all-cause death or readmission for cardiovascular cause in our rather small collective. As suggested by the observations made for natriuretic peptides, the prognostic value of congestion markers might change, probably increase, with the time point of assessment during the course from admission to discharge of an ADHF hospitalization to the following chronic stable phase. Supporting this hypothesis, IVCmax has shown to be highly predictive for an adverse outcome in patients with chronic heart failure [21].

Dyspnea is one of the most common symptoms in ADHF and its relief is associated to the patient’s general well-being [37]. Furthermore, in post-hoc analyses of ASCEND-HF (*n* = 7141 patients) and Pre-RELAX-AHF (*n* = 232 patients), it has been shown that dyspnea improvement may be a marker of decongestion and is associated with a better 30 day outcome [6, 38]. Dyspnea assessment by means of a DVAS is influenced by many factors, e.g., pulmonary comorbidities [38, 39]. In our population, the DVAS was not associated with the combined endpoint of all-cause death or readmission for cardiovascular cause.

Single signs of clinical congestion lack sensitivity as well as specificity of hemodynamic congestion [14]. Therefore, investigators combined several clinical congestion signs to congestion scores to improve the diagnostic value. For example, the EVEREST score was derived from the EVEREST trial population (2061 patients with LVEF < 40%, NYHA ≥ III, and two or more signs of volume overload) and was associated with an increased 30 day and all-cause mortality [11]. Combining clinical congestion signs to a clinical congestion score similar to the EVEREST score was not associated with the all-cause death or readmission for cardiovascular cause in our analysis. However, large-scaled registry analysis of more than 120,000 patients has previously shown that the severity of peripheral edema at presentation is associated with mortality during the index hospitalization as well as during follow-up [40]. Novel methods like the near-infrared spectroscopy for the assessment of the right atrial pressure might offer more precise tools in the future as compared to the crude semi-quantitative bedside assessment of jugular vein distention [41]. Even though all studied markers are meant to be associated with congestion, we did not observe a statistical correlation between these continuous variables. Congestion was formally defined as elevated left ventricular end-diastolic pressure in the past [15]. However, volume overload—as a consequence of renal sodium and water retention with resulting expansion of plasma value as well as extracellular interstitial water—plays an important role in heart failure-related congestion [42]. The lack of correlation between NT-proBNP, IVCmax, DVAS, and the clinical congestion score is probably caused by the fact that each parameter measures different aspects of congestion [43]. The NT-proBNP concentration increases in response to stretch of the cardiac wall due to transmural pressure and volume overload [44], rendering it a good marker of intravascular congestion and hemodynamic congestion [45]. The IVC diameter is likewise a gauge of intravascular congestion but values of both parameters are modulated by different parameters. Dyspnea measured on the DVAS is secondary to tissue congestion of the lung and the clinical congestion scores combine measures of tissue as well as intravascular congestion. This highlights that the investigated markers are not interchangeable but probably complemental. The authors of a recent position paper recommend integration of the degree of congestion assessed by multiple markers to evaluate euvolemia/congestion before discharge [9, 17]. Our findings clearly support this recommendation. However, a multimodal approach like this has not been evaluated in a randomized controlled trial so far. Moreover, it is unclear whether congestion markers are only of prognostic value or can be used to guide decongestion with the goal of improving prognosis. First data of the ESCAPE trial and PRIMA II trial suggest that tailoring treatment on a single congestion marker may not be beneficial for patients [4, 24]. The results of the ongoing CAVA-ADHF trial will answer the question whether IVC diameter-guided decongestion improves the level of decongestion at discharge or not [46].

### Limitations

Our study has several limitations that need to be acknowledged. First, all patients were enrolled in a prospective registry conducted in a single tertiary care academic medical center by a single investigator (SH) based on a standardized protocol, which may limit generalizability. Second, the analyzed population is relatively small, which may have an impact on the statistical power to detect significant correlations or outcome associations. However, the observed correlation coefficients are far from being significant which make it very unlikely that they would be relevantly different in a larger population.

## Conclusion

We observed no correlation between the degrees of congestion assessed by different continuous congestion markers. Only NT-proBNP determined at discharge was significantly associated with the composite endpoint of all-cause death or readmission for cardiovascular cause. Our findings indicate that the studied congestion markers reflect different aspects of congestion. Therefore, using several congestion markers in an integrative approach as recently recommended might be beneficial. However, randomized controlled trials are necessary to assess whether such an approach would improve the outcome of ADHF patients.

## Supplementary Information

Below is the link to the electronic supplementary material.

Supplementary file1 (DOCX 18 KB)
