# Supplementary material for: Lack of correlation between different congestion markers in acute decompensated heart failure
Source: Clin Res Cardiol. 2022 Jun 1;112(1):75–86. doi: 10.1007/s00392-022-02036-9 (PMC9849150; doi:10.1007/s00392-022-02036-9)
Supplement: Supplementary file 1 — Supplementary file1 (DOCX 18 KB) [file 392_2022_2036_MOESM1_ESM.docx]

**Online Table 1**

|  | estimate ± standard error for log(LOS) | P | FMI |
| --- | --- | --- | --- |
| **Admission** |  |  |  |
| NT-proBNP, per ln ng/l | 0.075 ± 0.062 | 0.225 | 0 |
| IVCmax, per mm | 0.029 ± 0.013 | 0.025 | 0.025 0.004 |
| CS, per point | 0.105 ± 0.040 | 0.008 | 0.012 |
| VAS, per cm | 0.021 ± 0.024 | 0.394 | 0.034 |
